# Supplementary material for: Drug repurposing for aging research using model organisms
Source: Aging Cell. 2017 Jun 16;16(5):1006–15. doi: 10.1111/acel.12626 (PMC5595691; doi:10.1111/acel.12626)
Supplement: Supplementary file 7 — Data S1 Zip‐Archive of all report cards. [file ACEL-16-1006-s007.zip › RC_Z70.pdf]

# Z70

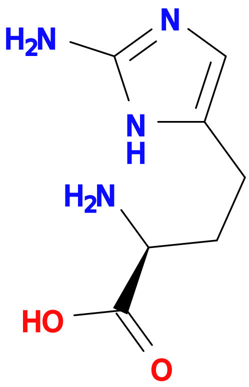


**Database identifiers**

| | |
|---|---|
| ChEMBLCompound | CHEMBL1099168 |

## Ranking

| | Rank | Score |
|---|---|---|
| Drosophila | 671/697 | 0.078 |
| C. elegans | NA | NA |

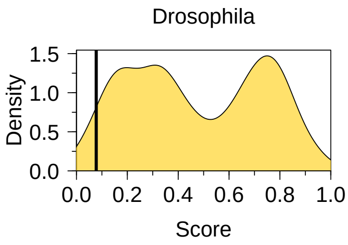


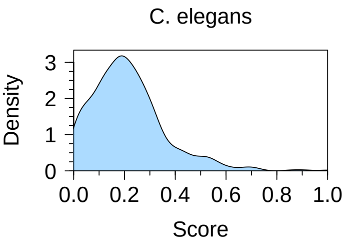


| | Ageing implication | Domain conservation | Binding site conservation | Binding affinity | Bioavailability | Lipinski | Promiscuity | Purchasability | Drug approval | **Total** |
|---|---|---|---|---|---|---|---|---|---|---|
| Drosophila | 0.624 | 0.802 | 1.0 | 0.173 | (0.9) | 0.0 | -0.0 | 0.0 | 0.0 | 0.078 |
| C. elegans | NA | NA | NA | NA | NA | NA | NA | NA | NA | NA |

## Names

No synonyms found

## Roles

ChEBI entry None has no roles

## Status

| | |
|---|---|
| Approved drug (according to ChEMBL) | No |
| Number of Rule of 5 violations | 0 |
| Binding affinity to original target in log units (RF-Score prediction) | 3.44 |
| Burns *C. elegans* bioavailability prediction | -4.14 |

## Compound Target Characteristics

### Arginase-1

Best gene implication in ageing for this target family came from gene P07824 via mapping the annotation from RGD 2150 annotated in RGD 2014-03-11. Annotation GO 7568 (aging) was Inferred from Expression Pattern

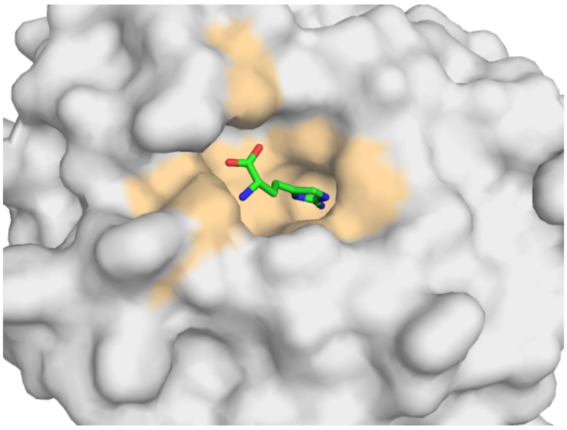

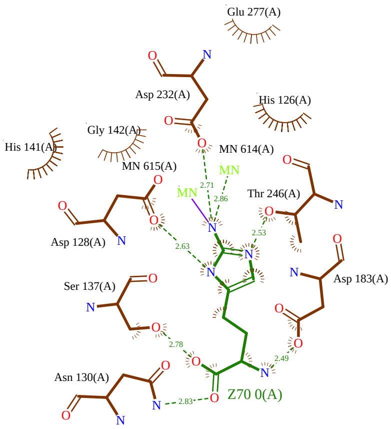


| protein | amino acids contacts (binding site) |
|---|---|
| PDB:3mfv:chainA:P05089 | D H D N S H G D E D D T E |
| sp:P05089:ARGI1_HUMAN | D H D N S H G D E D D T E |
| sp:P07824:ARGI1_RAT | D H D N S H G D E D D T E |
| sp:Q61176:ARGI1_MOUSE | D H D N S H G D E D D T E |
| tr:Q9NHA5:Q9NHA5_DROME | D H D N S H G D E D D T E |
| tr:O76895:O76895_DROME | D H D N S H G D E D D T E |
| sp:P00812:ARGI_YEAST | D H D N S H G D E D D T E |

| | whole protein | | domain-based | | contact-based | |
|---|---|---|---|---|---|---|
| protein | ident | simil | ident | simil | ident | simil |
| PDB:3mfv:chainA:P05089 | 1.0 | 1.0 | 1.0 | 1.0 | 1.0 | 1.0 |
| sp:P05089:ARGI1_HUMAN | 1.0 | 1.0 | 1.0 | 1.0 | 1.0 | 1.0 |
| sp:P07824:ARGI1_RAT | 0.87 | 0.96 | 0.87 | 0.96 | 1.0 | 1.0 |
| sp:Q61176:ARGI1_MOUSE | 0.87 | 0.96 | 0.87 | 0.96 | 1.0 | 1.0 |
| tr:Q9NHA5:Q9NHA5_DROME | 0.35 | 0.71 | 0.36 | 0.74 | 1.0 | 1.0 |
| tr:O76895:O76895_DROME | 0.34 | 0.7 | 0.36 | 0.74 | 1.0 | 1.0 |
| sp:P00812:ARGI_YEAST | 0.39 | 0.71 | 0.4 | 0.72 | 1.0 | 1.0 |

**arg (FBgn0023535) associated phenotypes**

partially lethal - majority die, some die during pupal stage

(Information from FlyBase)

**arg (UniProt:Q9NHA5) annotation**

**Cofactor**: Mn(2+) Evidence=(RuleBase:RU361159); Note=Binds 2 manganese ions per subunit. (RuleBase:RU361159);

**Pathway**: Nitrogen metabolism; urea cycle; L-ornithine and urea from L-arginine: step 1/1. (RuleBase:RU361159).

(Information from UniProt)

**arg (UniProt:O76895) annotation**

**Cofactor**: Mn(2+) Evidence=(RuleBase:RU361159); Note=Binds 2 manganese ions per subunit. (RuleBase:RU361159);

**Pathway**: Nitrogen metabolism; urea cycle; L-ornithine and urea from L-arginine: step 1/1. (RuleBase:RU361159).

(Information from UniProt)
